# Comparing Hip Dysplasia in Dogs and Humans: A Review

**DOI:** 10.3389/fvets.2021.791434

**Published:** 2021-12-15

**Authors:** Koen Willemsen, Michelle M. Möring, Netanja I. Harlianto, Marianna A. Tryfonidou, Bart C. H. van der Wal, Harrie Weinans, Björn P. Meij, Ralph J. B. Sakkers

**Affiliations:** ^1^3D Lab, Division of Surgical Specialties, University Medical Center Utrecht, Utrecht, Netherlands; ^2^Department of Orthopedics, University Medical Center Utrecht, Utrecht, Netherlands; ^3^Department of Clinical Sciences, Faculty of Veterinary Medicine, Utrecht University, Utrecht, Netherlands; ^4^Department of Biomechanical Engineering, Delft University of Technology, Delft, Netherlands

**Keywords:** hip dysplasia, one health, treatment, translational, comparative, acetabulum

## Abstract

Hip dysplasia (HD) is common in both humans and dogs. This interconnection is because humans and dogs descended from a common ancestor and therefore have a similar anatomy at micro- and macroscopic levels. Furthermore, dogs are the animals of choice for testing new treatments for human hip dysplasia and orthopedic surgery in general. However, little literature exists comparing HD between the two species. Therefore, the aim of this review is to describe the anatomy, etiology, pathogenesis, diagnostics, and treatment of HD in humans and dogs. HD as an orthopedic condition has many common characteristics in terms of etiology and pathogenesis and most of the differences can be explained by the evolutionary differences between dogs and humans. Likewise, the treatment of HD shows many commonalities between humans and dogs. Conservative treatment and surgical interventions such as femoral osteotomy, pelvic osteotomy and total hip arthroplasty are very similar between humans and dogs. Therefore, future integration of knowledge and experiences for HD between dogs and humans could be beneficial for both species.

## Introduction

**Figure 1:**
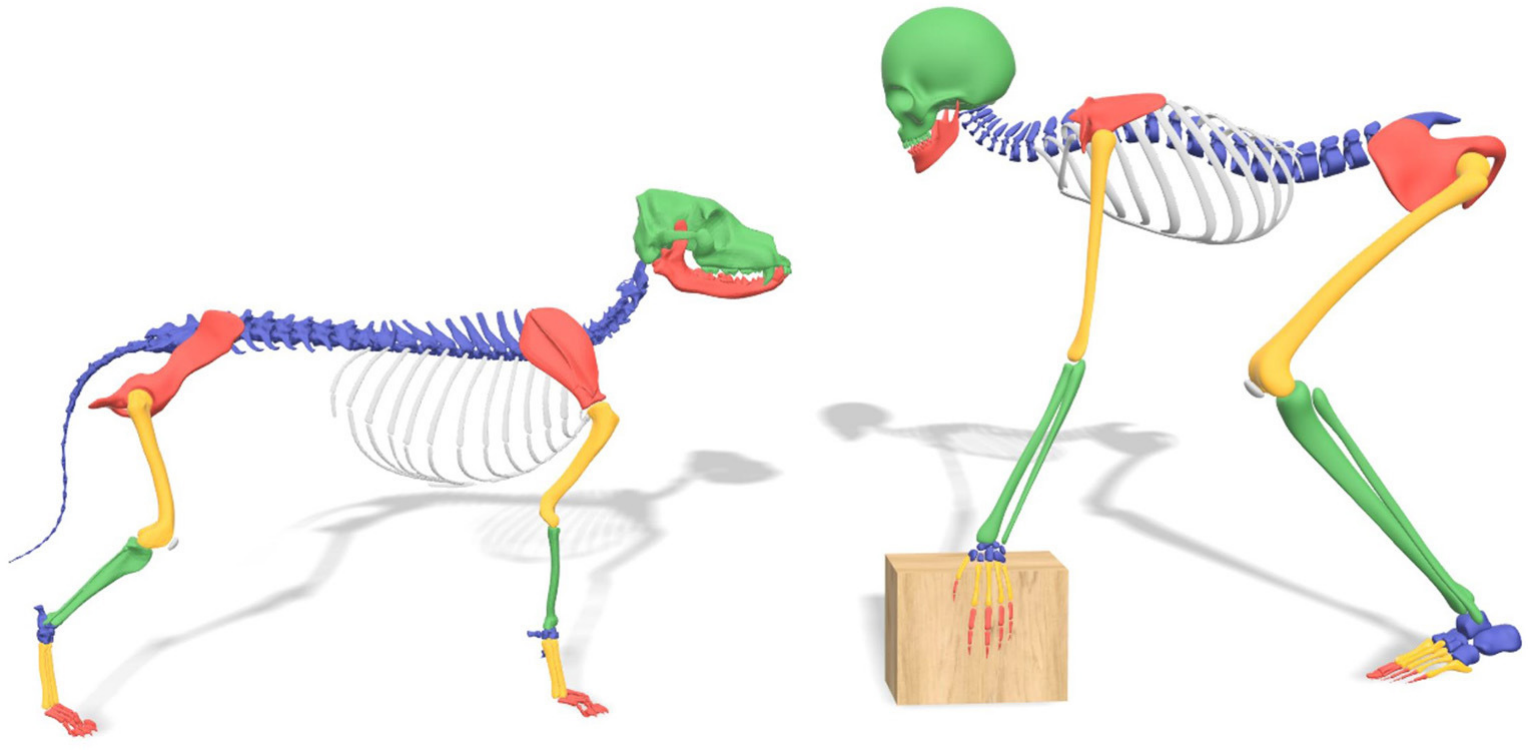
Comparison skeletal structure between dog and human.

Dogs and humans have developed from a common ancestor. Both species are vertebrates and terrestrial mammals, with a very similar homologous musculoskeletal structure (Figure 1). Because of this resemblance in body structure, certain diseases in both species have a common ground. One of these diseases is hip dysplasia (HD). HD was first described in dogs in the 1930's (1) and in humans as early as Hippocrates (2). HD is better known as canine hip dysplasia (CHD) in dogs, and developmental dysplasia of the hip (DDH) in humans. The prevalence of HD in humans varies between 0.1and 10%, depending on the population and definition (3, 4). In dogs, the prevalence varies between 0 and 73.4%, depending on the breed (5–8).

There are similar characteristics for HD in humans and dogs. In both species the acetabular cover of the femoral head is insufficient, either because the acetabulum (5, 9, 10) or the femoral head (5, 9, 10) is deformed, or joint laxity (2, 5) is present. This disturbed femuro-acetabular relationship causes abnormally high peak forces (1, 6, 10) with or without joint instability and (sub)luxation (2, 5, 9) resulting in osteoarthritic changes (2, 5, 9). The body tries to counter the sequela of HD in both species by thickening and stiffening of the joint capsule (10–12) in order to reduce the laxity (11, 12). However, HD will eventually induce osteoarthritis (OA) resulting in pain (6, 13), lameness (14), and loss of limb function (6, 13), reducing quality of life.

While CHD and DDH show numerous similar characteristics, disease management is not always the same for both species. In this review we give an overview of the anatomy, etiology, development, diagnostics and treatment of HD in humans and dogs. This will provide veterinarians and physicians a perspective and incentive to share the combined translational knowledge.

## Anatomy

Initially, the anatomy of humans and dogs may seem very different. For instance, an obvious difference between dogs and humans is that dogs have a quadruped (four-legged) gait while humans have adopted a bipedal (two-legged) gait. While some anatomical differences have developed due to this difference in gait, dogs and humans have more in common than one might think (Figure 1).

The human biped gait has a smaller base of support (less point of ground contact) and an elevated center of mass (15, 16). To balance the body, humans have developed a lumbar lordosis so the center of mass (head, arms and trunk) is directly above the point of ground contact. This is also more energy efficient (17, 18). Similarly, a wider pelvis with more laterally oriented iliac crests (as opposed to the coronal plane in dogs) allowed for some changes in musculature, improving balance on one leg, energy efficiency, and increasing stride length (16–18).

While the load orientation (19) of the hip is very similar in dogs and humans, the difference between biped and quadruped gait gives different load distribution between limbs. Humans distribute their bodyweight between two legs while dogs distribute their weight over four legs, with the front legs carrying approximately 60–65% of the bodyweight (20–22). Because of the dominance of the front legs over the hind legs, dogs are capable of compensating for hip abnormalities (e.g., HD) by lowering their neck and increasing the load on the non-affected side (20). Humans can also reduce load on the affected sides by using instruments such as a cane or a stroller (23).

The canine and human anatomy is not just similar on a macroscopic level. Human and canine hips have a similar cortical microstructure (24, 25) and long bone vascularization (25, 26). Because of these external and internal similarities, the dog has long since been (one of) the animal(s) of choice for orthopedic research aimed at humans (24, 27, 28).

## Etiology and Pathogenesis

While the exact etiology of HD for both humans and dogs remains unknown (10, 29), the general agreement is that both genetic and environmental factors influence the development of CHD and DDH (4, 8). First the genetic factors are discussed, followed by environmental factors and finished with the pathogenesis.

Some examples of common genetic factors that influence the occurrence of HD in both species include breed (1, 6) ethnicity (29), increased anteversion angle of the femur (2, 30, 31), neck shaft angle of the femur (2, 31), and collagen composition (1, 4). Because of these high genetic factors, family anamnesis is important for discovering HD in humans and improving breeding programs in dogs. However, not all genetic factors have an known influence on both species, e.g., a clear genetic factor such as female sex in humans is known for a higher incidence of HD (4:1, Female:Male) (3, 9), while no such relation is known for dogs (8, 32).

Besides genetic factors there are many different environmental factors influencing the development and incidence of HD. Some common environmental factors concern the nutritional state such as diet (33), obesity (14, 34, 35) and high birth weight (5, 36). Furthermore, environmental factors such as to seasonal influence (4, 7) and hormone levels have an association with HD (4, 37). Other environmental factors concern disturbances of the biomechanical equilibrium in the pelvic area, e.g., transitional vertebrae (38) can change the forces flowing through the hip. This also happens with restrictive swaddling of babies which is common in certain human cultures. Swaddling limits the abduction and therefore reduces the required force on the triradiate growth plate (39, 40).

Most of previous mentioned factors are identical between both species, however factors surrounding birth do differ between species. Humans have only one baby at a time, while dogs have several pups in their litter, meaning intra-uterine mechanical factors are different. For instance, breech presentation in humans is associated with a high incidence of DDH in single child pregnancies, but no relation has been found in twin pregnancies (3, 41). Similarly, other factors like oligohydramnios (42), breach position, being first born (3, 4) and even the preference for the left hip are commonly described in humans, but not in dogs who are typically born in a litter. The preference for the left hip in humans might be explained as the left hip is often positioned against the mother's spine in the womb, which limits abduction (5, 9) and reduces force on the developing triradiate cartilage.

Besides genetic and environmental factors, there is a clear developmental aspect in both DDH and CHD. Both species need the femoral head to be centered on the triradiate cartilage of the acetabulum in order to develop normal joint morphology (5, 9, 10). Well balanced supporting structures of the joint like the pelvic muscles (2, 43), the joint capsule, and the femoral head ligament are important to maintain joint congruity (5). A larger amount of pelvic muscle mass is associated with a lower incidence of CHD (2, 10). Similarly, weak pelvic muscles in dogs are associated with adverse joint changes (2). For humans, weak pelvic muscles have also been theorized to cause dysplasia and degenerative joint change (43).

Human newborns with normal hips might develop HD later in life (44). Of newborns with perceptible HD, 88% will develop into normal hip joints by the age of 8 weeks, without any intervention (9, 39, 45). However, the older the infant is, the less likely it will be that natural normalization occurs (9). The abnormal stress on the hip joint caused by HD can cause pain even before degenerative changes start. Patients with HD can already present with OA in adolescents and young adults (46). In CHD, the hips are typically normal at birth (2, 10). However, early signs such as edematous and slightly torn ligaments of the femoral head can already be seen around 4 weeks of age (47, 48). Subsequently, further dysplastic joint changes develop such as joint laxity and deformity of the acetabulum and femur (47). This deformity eventually leads to cartilage changes, pain and lameness. Some dogs start showing clinical signs around 3–12 months of age (10, 49), while other dogs remain asymptomatic and present long after full maturation.

## Diagnosis

Early detection of HD in humans and dogs can lead to earlier interventions, which is important for disease management (9, 50). To ensure early detection in humans, many countries have developed and implemented screening programs aimed at diagnosing DDH in infancy (51). In dogs early detection of HD is usually driven by the occurrence of clinical signs from age of 4 to 5 months, which will stimulate owners to seek veterinary advice for diagnostic testing, usually with radiography. However, screening programs for CHD in dogs are recommended for breeding, and is globally implemented. However, the minimum age for screening using radiographs is commonly set at skeletally mature age of 1 year for most breeds and at 18 months for selected large to giant breeds. Since HD in young dogs is commonly asymptomatic this will prevent early detection of HD in dogs. While the details might differ, the clinical diagnostics in dogs and humans are very comparable, generally consisting of physical examination and imaging.

### Physical Examination

Early observational findings during physical examination in humans are restricted abduction and difference in leg length in case of hip (sub)luxation (9, 39). Asymmetric gluteal folds who were once thought to be of high clinical significance did not have a high predictive value and are therefore not used anymore (52). In a child of walking age the Trendelenburg sign can be seen with or without asymmetries, like a proximal thigh crease, posterior knee crease, wide perineum, prominent hip curvature, and limping (39, 53). With bilaterally affected hips this asymmetry is usually absent, but bilateral Trendelenburg sign, waddling gait (9, 39) and bilateral limited abduction (9) can be seen. Dogs should be observed in rest, during activity, and after exercise (54). The main finding in young dogs with hip joint laxity is lameness that increases during exercise (1), but also hip atrophy, reduced range of motion and pain during flexion and extension may be present. Hip pain in dogs is usually noted by abnormal behavior like bunny hopping with pelvic limbs, difficulty to rise, and less playfulness together with grunting, whimpering, or whining (55). The combined pain assessment by both the owner and the veterinarian seems to work best (55), but there is no consensus on a gold standard (1, 55). Furthermore, dogs do not need a pain free full range of motion for a normal gait (11), typically dogs with no or minimal clinical signs could have severe dysplastic hips (12).

**Figure 2:**
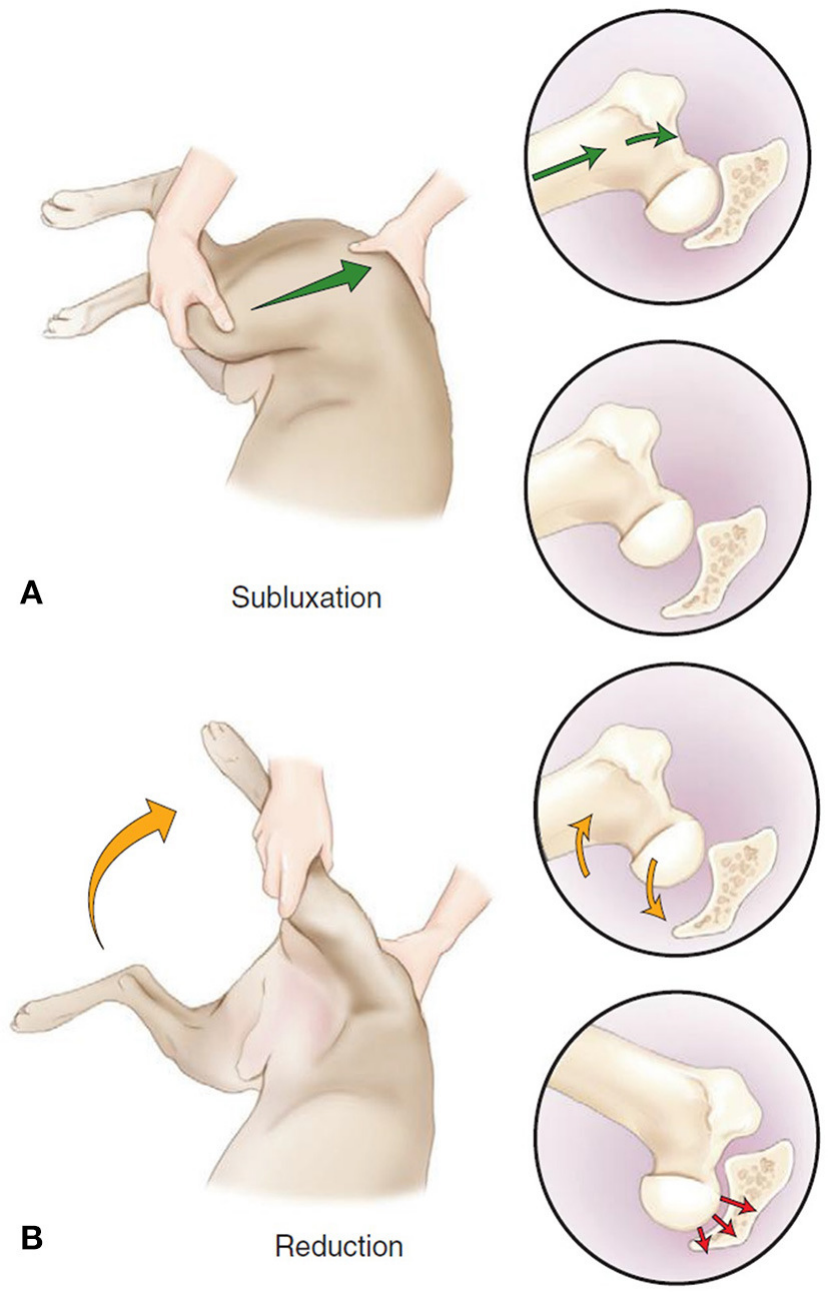
The Barlow and Ortolani test in dogs. **(A)** “Barlow” (subluxation) test. The dog is positioned in lateral or dorsal recumbency. In lateral recumbency, the examiner is caudal to the dog with one hand on the distal stifle (flexed to 90 degrees) and the other is dorsal to the pelvis, with the thumb resting over the greater trochanter. The limb is in an adducted position, and force is applied toward the dorsum of the dog up through the femur (green arrow), causing dorsal subluxation in a hip with joint laxity. **(B)** Ortolani (reduction) test. The limb is slowly abducted (yellow arrow) while force along the axis of the femur is maintained. A positive Ortolani sign is felt when a click or clunk is heard or palpated as the subluxated femoral head reduces into the acetabulum (red arrow). Figure reproduced without modification from (56) under the Creative Commons Attribution 4.0 International License ().

**Figure 3:**
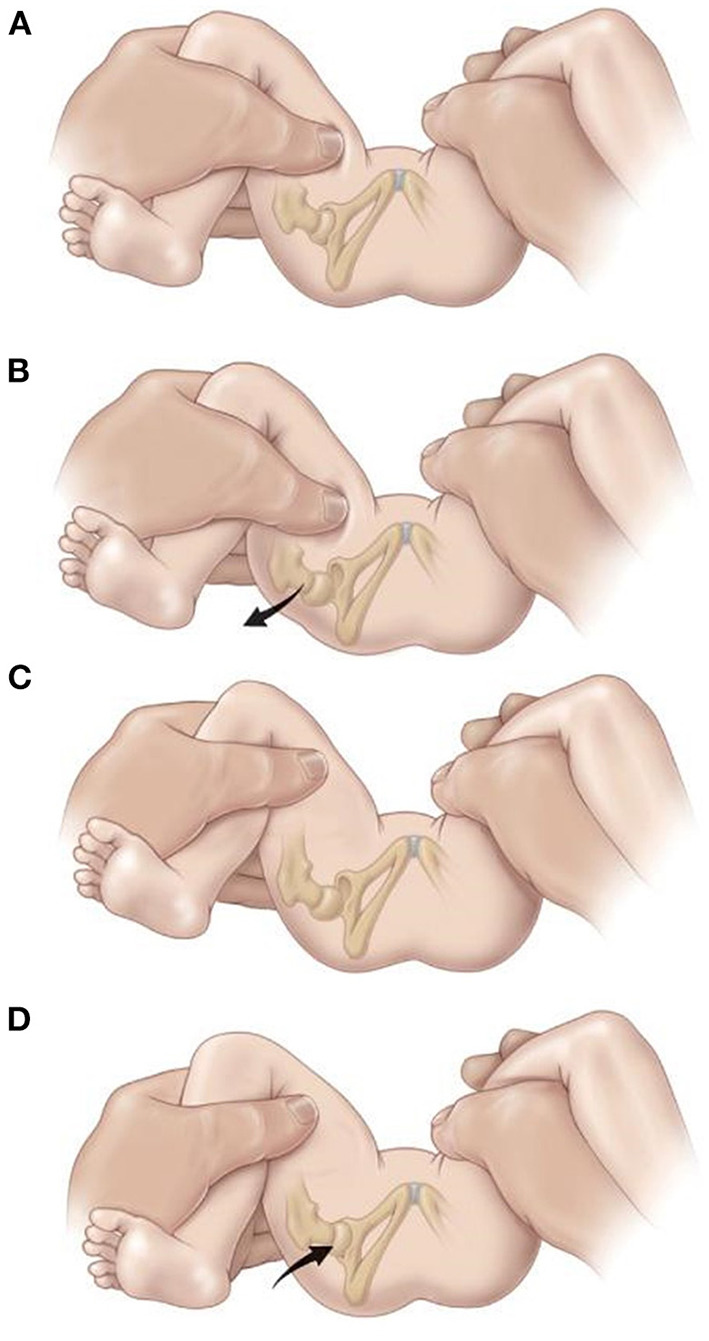
The Barlow and Ortolani test in Humans. The Barlow test for developmental dislocation of the hip in a neonate. **(A)** With the infant supine, the examiner holds both of the child's knees and gently adducts one hip and pushes posteriorly. **(B)** When the examination is positive, the examiner will feel the femoral head make a small jump (arrow) out of the acetabulum (Barlow's sign). When the pressure is released, the head is felt to slip back into place The Ortolani test for developmental dislocation of the hip in a neonate. **(C)** The examiner holds the infant's knees and gently abducts the hip while lifting up on the greater trochanter with two fingers. **(D)** When the test is positive, the dislocated femoral head will fall back into the acetabulum (arrow) with a palpable (but not audible) “clunk” as the hip is abducted. [Reprinted with permission from Tachdjian's Pediatric Orthopedics (57), Elsevier Publishing].

For examining the depth of the acetabulum and joint laxity, the following clinical tests are performed: the Barlow test, the Barden test, the Galeazzi test, and the Ortolani test, all of which were originally developed for use in humans (1, 9). The Ortolani test is most commonly used in both humans (9, 51) (Figure 2) and dogs (1, 54) (Figure 3). The Barlow test is also commonly used in dogs (1, 54). It should be noted that while on human infants and dogs these tests can be directly performed, these tests often require sedation or general anesthesia when dogs are not cooperative (54) (Figures 2, 3).

Since DDH and CHD develop at different rates, a positive result has slightly different implications. In humans a positive result indicates subluxation or dislocation of the femoral head typically due to decreased coverage (9, 51). A positive Ortolani test in young dogs usually points to joint laxity (1, 54) which is a sign of HD in development (58).

### Imaging

**Figure 4:**
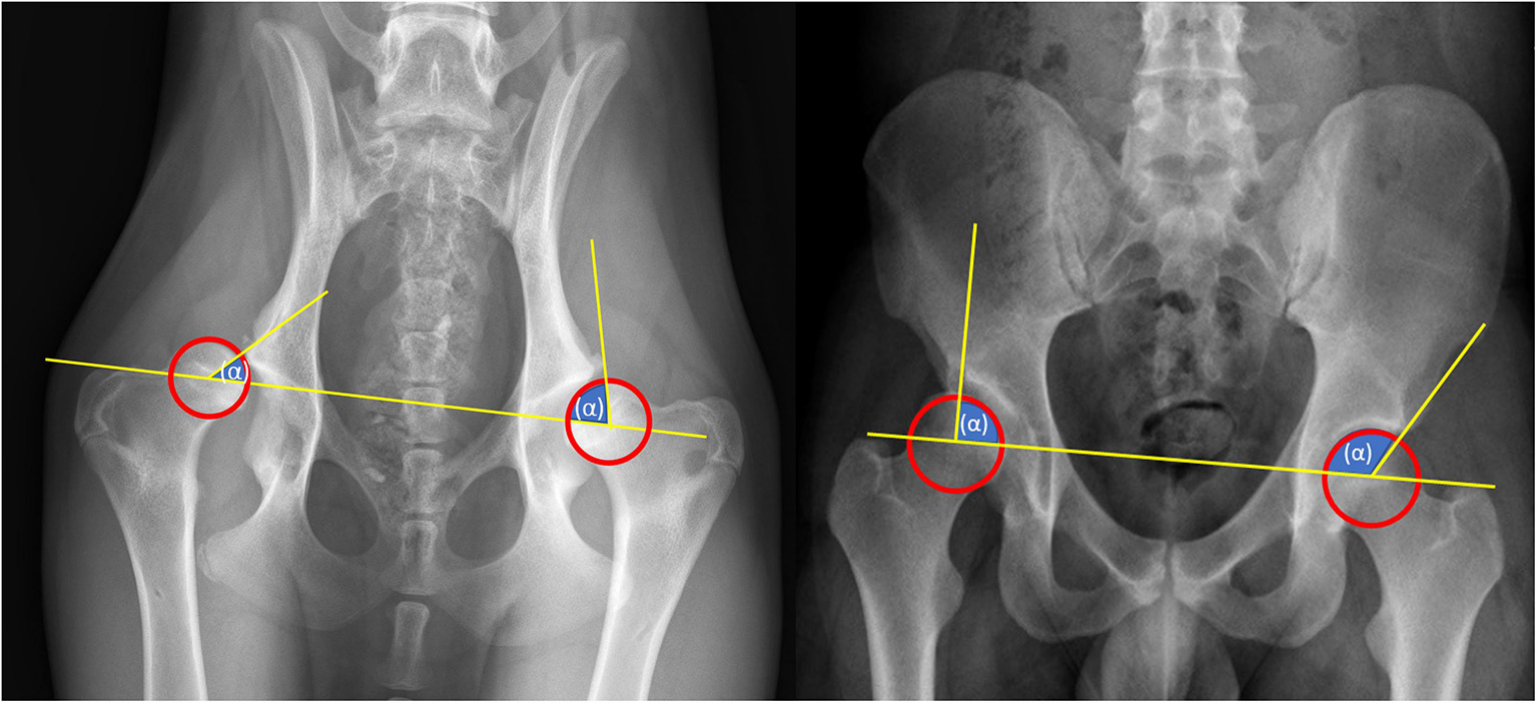
Radiographic diagnostics. Left: Norberg-Angle, take the center of each femoral head (hip ball) and draw a line between them. Then take the center of the femoral head and draw a line to the outer point of the pelvis. The angle between these lines is the Norberg angle. The Norberg angle is calculated for each hip joint. A normal Norberg Angle ranges from 100/105 to 115 degrees. <100 degrees is dysplastic. Right: CE-angle, take the center of each femoral head (hip ball) and draw a line between them. Then take the center of the femoral head and draw a line to the outer point of the pelvis. The angle between these lines is the CE angle. The CE-angle is calculated for each hip joint. A normal CE Angle ranges from 110/115 to 130 degrees. <110 degrees is dysplastic.

Radiography is the golden standard for diagnosing HD in dogs (1). Historically, pelvic and hip radiography has been used for diagnosing HD in humans (9, 59). However, in some parts of the worlds X-rays have been partially replaced by ultrasound imaging for young patients (60) as classification of HD on X-rays is currently considered less reliable before ossification of the femoral head center occurs at 4–6 months (9, 39). Although there are widely accepted ultrasound classifications, ultrasound images still has drawbacks, such as: high variability and low agreement (61). In dogs the ossification starts at 8 weeks, which makes ultrasound less useful as the ossification distorts the view of the acetabulum on ultrasound (54). The way radiographs are attained and measured is remarkably alike, both in dogs and humans the radiographs are taken in ventrodorsal and anterior-posterior position to measure the center-edge (CE)-angle and the Norberg angle (Figure 4)(59, 62–64). Besides the CE-angle and the Norberg angle, other radiographic parameters can be measured to increase the validity of the diagnosis. However the CE-angle is the most renown (65).

## Treatment

The available treatments for HD in humans and dogs change when patients develop toward skeletal maturity (39). Young patients have soft and pliable bone with good remodeling capabilities due to growth. Therefore, HD treatment can focus on stimulating growth by redirecting the femoral head to the center of the triradiate growth plate of the acetabulum in order to create a stable well-covered hip joint (9). As the patient matures the growth potential of bone decreases and the ability to correct the joint relationships with it. When forming a congruent well-covered joint is no longer an option, osteoarthritis might develop.

Skeletal maturity is reached around 15–18 years in humans (43) and 1–1.5 years in dogs (66). The triradiate cartilage (acetabular growth plate) closes around 14 years of age in humans (67) and around 6 months of age in dogs. There is no clearly defined separation between treatment options for certain ages and stages of bone development. Therefore, in order to accommodate this review a separation is made between “early” and “late” treatment.

### Early Non-surgical Treatment

Early treatment of hip dysplasia in humans distinguishes between a (sub-)luxated and a non-luxated hip. A luxated hip needs repositioning first before the acetabular dysplasia can be treated.

When a dysplastic hip is diagnosed with (sub)luxation of the femoral head, a Pavlik harness is most often applied as first treatment. The Pavlik harness uses several straps to flex the hips and knees and prevent adduction, while movement is still possible (9, 68). In a child treated within the 1 weeks after birth this position forces the femoral head into the acetabular socket and onto the triradiate cartilage. After creation of a stable joint, the harness is still worn for 23 h per day until a morphologically normal hip joint is found on imaging (39, 68). When a stable reduction of the hip is not reached within 3–4 weeks, reposition of the hip under full relaxation under anesthesia might be tried, with or without adductor tenotomy, followed by a plaster cast usually for 3 months (9, 68). If repositioning of the hip fails under anesthesia, open reposition of the hip should follow, usually after the age of 6–9 months (9). There is no consensus about optimal treatment length (9, 69). The Pavlik harness has not been described for dogs, since they do not easily accept external hip coaptation devices. However, a somewhat similar concept was used in puppies with genetic predisposition for CHD that were raised in a small cage (1 m^3^) until they finished growing. This caused them to sit more often with their hind limbs spread (flexion and abduction) and reduced the prevalence of CHD. This method prevents dogs from socializing and is therefore not used in daily practice (48, 54).

In young dogs with CHD that start to show clinical signs, usually from age 4 to 5 months, the non-surgical treatment measures are similar to those at older age and therefore will be discussed in more detail in section Late Non-surgical Treatment.

### Early Surgical Treatment

If non-surgical treatments are ineffective or the child gets older than 9–18 months, open reduction of the hip joint can be performed (9, 46). Open reduction focuses on reducing the subluxated or dislocated hip and creating a stable hip joint, similar to closed reduction. Open reduction of the hip is usually combined with capsular reefing and the release of the transverse acetabular ligament, and may be combined with an acetabular or femoral osteotomy in order to create a stable well-centered hip (9). After the open reduction, the child is treated with a spica cast to maintain the position of the hips (9). In dogs open reduction for a luxated hip due to severe HD is never performed. Hip luxation in young dogs with HD, called the luxoid hip, is usually an indication for early euthanasia, femoral head and neck resection or total hip replacement from age 7–9 months.

In the older child with residual hip dysplasia, an acetabuloplasty, e.g., the Dega, or Pemberton acetabuloplasty is commonly used to improve centering and acetabular coverage of the femoral head (70, 71). While both procedures are different, both are curved partial osteotomies of the ilium, with a small (bone) graft placed in the osteotomy. This partial osteotomy causes a hinging effect in the horizontal line of the triradiate cartilage and will reshape the acetabulum, reducing its diameter, yet increasing depth (72). The Pemberton acetabuloplasty improves anterior and lateral femoral head coverage, but not coverage of the posterior femoral head. The Dega acetabuloplasty increases the anterior, lateral, and posterior femoral head coverage (72). Acetabuloplasties give the best results when used on patients 2–8 years old (46).

Another common technique for pelvic osteotomies in young children is the Salter osteotomy (9, 72). This technique is based on a complete osteotomy of the ilium bone just superior of the acetabulum and redirection of the existing acetabulum (73, 74). Therefore, the Salter osteotomy does not alter the shape of the acetabulum. A possible complication described in the Salter osteotomy is instability (71, 74) and another complication for the Salter and Pemberton acetabuloplasty (71) is overcorrection, leading to excessive coverage of the femoral head resulting in femoral acetabular impingement (71, 74).

The majority of early surgical treatments, like the Pemberton and Salter osteotomy used in humans are not applicable in dogs simply because CHD is not detected early enough in the dog's life. The only comparable treatment in dogs is juvenile pubic symphysiodesis (JPS). The JPS is an early surgical treatment for CHD, and to our knowledge has not been used in humans. JPS is a relatively simple surgery in which the cartilage of the pubic symphysis is destroyed through electrocauterization. The heat causes the chondrocytes to become necrotic, resulting in premature closure of the pubic symphysis. Since other parts of the pelvis continue to grow, the acetabulum is rotated ventrolateral, similarly to the human Pemberton and Salter osteotomy, which allows for greater femoral head coverage (12, 54, 66). To be effective, JPS should be performed before week 18 in small dogs or week 22 in large breed dogs (12, 66).

Osteotomies of the femur are frequently used in humans and infrequently in dogs. In dogs aged ½−2 years the intertrochanteric femoral osteotomy is used to reduce the neck shaft angle (varisation) and anteversion angle, which are often increased in dysplastic hips. The femoral head is moved more medially (12, 75, 76) which helps redirect the femoral head into the acetabulum (75, 76). This is achieved by removing a bone wedge from the proximal femur and the bone is then stabilized by a hook plate (12, 75, 76). In humans, a femoral osteotomy can be performed sub- or intertrochanteric. The osteotomy also aims to reduce the anteversion (also called derotational osteotomy) and neck-shaft angle. Femoral osteotomies in humans are often combined with open reduction and acetabular osteotomies, between the ages of 2–14 years (69).

### Late Non-surgical Treatment

There are various late non-surgical treatments for dogs and humans with hip dysplasia. To decrease pain (1, 77), reduce lameness (14), and delay onset of osteoarthrosis (1, 66) a variety of treatments are available including medication like NSAIDs (1), reducing body weight (34, 77), life style changes including training of pelvic muscles, exercise programs and the limiting sudden explosive movements (like throwing a ball for dogs). On average, weight loss in dogs delays surgery for another 3 years (10) and in overweight dogs and humans 10% body weight reduction is associated with a relieve in symptoms and signs (14, 77). Another non-surgical intervention is the nutraceutical market, which is especially big in the veterinary market. Nutraceuticals are food additives or supplements that are purported to have a disease modifying potential in hip dysplasia and osteoarthritis, but also other conditions. An example of a nutraceutical is Polysulfanated glycosaminoglycans (PSGAGs) which proposedly stimulates collagen synthesis and inhibits the breakdown of collagen (13) which may help reduce subluxation (54).

Another option for early non-surgical treatment is physiotherapy (78, 79). In both dogs and humans physiotherapy and hydrotherapy is an important component first as a conservative treatment option but also as an important aspect in post-surgical rehabilitation (78, 79).

### Late Surgical Treatment

**Figure 5:**
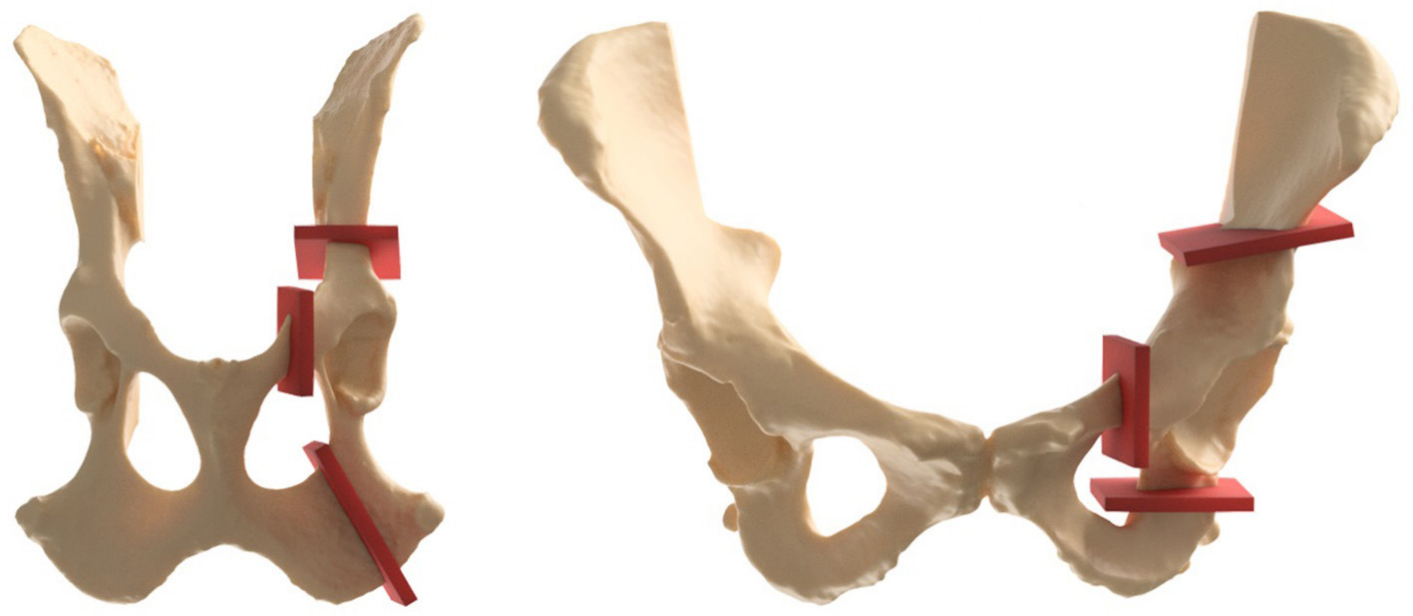
Canine left and human right triple pelvic osteotomies (purple planes) are made in the pubic, ischial and iliac bones and subsequently the acetabulum is rotated ventrally (dogs) or anteriorly (humans) to improve femoral head coverage and increase the load bearing area.

Originally designed for humans with HD, triple pelvic osteotomy (TPO) has also become a successful procedure for dogs with HD (12, 80)(Figure 5). This surgery can be used in young dogs (1, 54, 81), but more often in adolescents (46, 82, 83) and young adults (82, 83) without or with minimal degenerative joint damage (1, 49, 54, 83). In dogs, the surgery is preferably performed before full skeletal maturity is reached, while in humans it can be used both before and after the triradiate cartilage closes (9, 83). However, humans have more early surgical treatments available (e.g., Salter & Pemberton), deferring the more invasive TPO to older patients. Over the years there have been many changes in specific surgical techniques, but the general outline of TPO remains the same. Osteotomies are made in the pubic, ischial, and iliac bones, and the acetabulum is subsequently rotated ventrally to improve femoral head coverage and increase the load bearing area (80) (Figure 5). The acetabulum is then fixated in place by plates, screws, or K-wire. Clinical reduction of lameness after TPO, and improvement in weight bearing of 86–92% is reported in dogs (75, 76, 81). The joint laxity is reduced following TPO in dogs (80, 81), but degenerative changes cannot be stopped completely (81). In humans, TPO causes a long term reduction of pain and improvement of function (84) and a (total hip free) survival of 68% after 25 years is reported. Recently, dual pelvic osteotomy (DPO) has been recommended in dogs (75, 85), it has also been described in humans (72). DPO is similar to TPO, with a faster post-operative recovery, as there is no osteotomy of the ischium and therefore no pelvic discontinuity (75, 85).

Shelf arthroplasty is a commonly used salvage procedure for HD in humans (86). It involves the placement of an autologous bone graft outside of the joint capsule superior to the acetabulum (87, 88). The graft can be impacted into the bone or be held in place by a screw (88), improving the support structure of the joint (9). Capsular metaplasia causes the improvement of the articulating surface. The improved support and improved femoral head coverage helps improve the weight bearing surface (9) and delays the progression of OA. This procedure is preferably performed in younger patients with minimal arthritic changes, however it is mostly reserved as a salvage procedure as other treatments are not eligible. The survival of the shelf procedure can be up to 72% at 35 years of follow-up (86). A similar procedure called the biocompatible osteoconductive polymer (BOP) procedure has been described as an alternative to TPO in adolescent dogs (12, 75, 76). Instead of autologous bone graft, biocompatible osteoconductive fibers were used to increase coverage, because the fibers were expected to promote bony ingrowth (12, 75, 76). Despite that the shelf procedure was successful in humans, BOP in dogs never became a common procedure because of uncontrolled bone growth (75, 76). New procedures involving 3D-printed titanium shelfs (89) or 3D-printed biodegradable magnesium phosphate shelfs (90) are still being developed in dogs and when successful these procedures hopefully find their way back to the human clinic.

**Figure 6:**
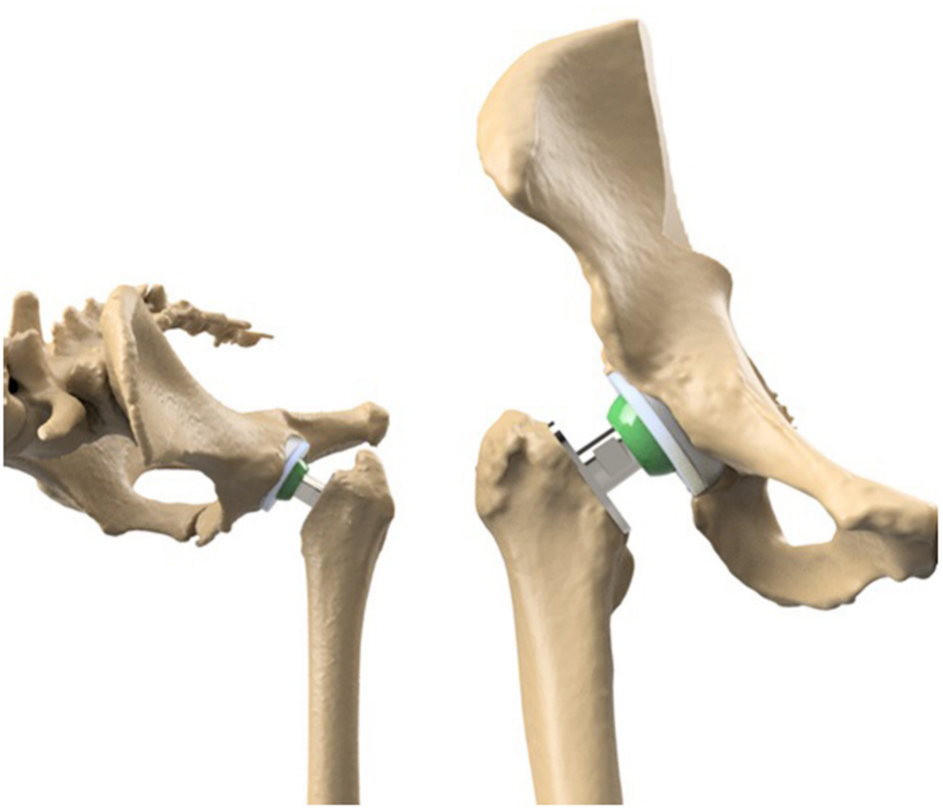
The comparable set-up of the total hip arthroplasty in humans and dogs. On the left the set-up in dogs and on the right the set-up in humans.

While being one of the most effective procedures, total hip arthroplasty (THA) in humans and dogs (12) is often postponed as the last treatment option (Figure 6). When young patients with a demanding lifestyle receive a THA they may need one or more revisions in their lifetime due to implant wear. However, every revision is more difficult to perform due to fibrosis in the perioperative area. Therefore, in humans, the need for THA is preferably postponed beyond the age of 60 to prevent revisions in the long term. Although THA has been available for dogs for three decades, it remains an expensive treatment option, especially when the owners have no insurance (49). Also, THA in humans can become technically demanding due to anatomical differences in dysplastic hips making it difficult to ream a large enough bony bed to support an acetabular cup (91). In dogs the procedure is technically demanding due to breed anatomic differences but can be used in dogs of any size or shape when aged 9 months or older (12).

It is good to note that implant improvements have benefited for cross species research. For example, due to the active nature of dogs, the THA materials demand is high and companies specializing in canine THA have benefited from the prosthetic knowledge being researched and developed for human medicine. For example, similar durable materials developed for human cups and stems are translated to the dog THA allowing dogs to perform without the need for revision beyond a decade lifetime, with a biomechanically demanding lifestyle asking for more cyclic loading of their implants than humans. Vice versa, in dogs new products are developed e.g., to decrease stem loosening, because dogs demand immediate full weightbearing after surgery due to there non-compliance to life style restrictions. One example of a successful concept in THA surgery in dogs (Zürich cementless THA) is the immediate stem screw fixation at the medial femoral cortex instead of press fit fixation (92). Likewise, in a few years more developments might be translated back from the veterinary to the human market.

One of the least performed in humans but most commonly executed salvage procedures in dogs is the femoral head and neck excision (75, 76). The surgery is relatively easy to perform and has low costs, and is most effective in dogs with low body weight (75, 93). The removal of the femoral head and neck leads to a fibrotic pseudoarthrosis (false joint), allowing for relative pain free movement (12, 49). Femoral head and neck excision generally has good results in relieving pain, however possible side effects are extensive rehabilitation, decreased range of motion, muscle atrophy and limping due to decreased limb length (12, 75, 76, 93). Important factors in the outcome are body size (12, 75, 76), dog temperament and activity (12, 94). This procedure performed in humans is called a “Girdle stone” procedure, but only as the last option (94).

## Conclusion

In this review we described the anatomy, etiology, development, diagnostics and treatment of HD in humans and dogs. Humans and dogs have similar anatomy on micro- and macroscopic levels. HD as an orthopedic condition has many overlying characteristics in humans and dogs in terms of etiology and pathogenesis. Likewise, treatment of HD shows many similarities. There is much parallel use of early and after growth (conservative) treatments and interventions. Moreover, many of the surgical treatments for HD that were developed for humans have first been tested in experimental dogs. Procedures that became successful in humans found their way to the veterinary field and are now commonly used in companion animal clinics. We suggest that further exchange between research on HD in humans and dogs can be beneficial for the treatment of HD in humans and dogs.

## Author Contributions

BW, BM, RS, HW, and KW: conceptualized the study. KW, MM, and NH: collected the data. KW, MT, MM, NH, and RS: analyzed the data. HW, BM, MT, and BW: supervised the project. KW, MT, RS, NH, MM, HW, BM, and BW: wrote, edited and reviewed the paper. All authors have read and approved the final submitted manuscript.

## Funding

KW and HW report a Governmental research grant from Interreg VA Flanders—The Netherlands program, during the conduct of the study; KW and HW report grants from the Smart industry project 15479 funded through governmental grant by NWO domain TTW, during the conduct of the study; MT, HW, and BM report funding from the Dutch Arthritis Society (LLP12/22). The funding sources played no role in the study design, data analysis and interpretation, nor drafting of the manuscript or the decision to submit it for publication.

## Conflict of Interest

The authors declare that the research was conducted in the absence of any commercial or financial relationships that could be construed as a potential conflict of interest.

## Publisher's Note

All claims expressed in this article are solely those of the authors and do not necessarily represent those of their affiliated organizations, or those of the publisher, the editors and the reviewers. Any product that may be evaluated in this article, or claim that may be made by its manufacturer, is not guaranteed or endorsed by the publisher.
